# Development of a real-time clinical decision support system upon the web mvc-based architecture for prostate cancer treatment

**DOI:** 10.1186/1472-6947-11-16

**Published:** 2011-03-08

**Authors:** Hsueh-Chun Lin, Hsi-Chin Wu, Chih-Hung Chang, Tsai-Chung Li, Wen-Miin Liang, Jong-Yi Wang Wang

**Affiliations:** 1 Department of Health Risk Management, School of Public Health, China Medical University, 91 Hsueh-Shi Road, Taichung 40402, Taiwan; 2 Department of Medicine, School of Medicine, China Medical University, Taiwan; 3 Buehler Center on Aging, Health & Society and Department of Medicine, Feinberg School of Medicine, Northwestern University, Graduate Institute of Biostatistics, China Medical University, Taiwan; 4 Graduate Institute of Biostatistics& Biostatistics Center, China Medical University, Taiwan; 5 Department of Health Services Administration, School of Public Health, China Medical University, Taiwan

## Abstract

**Background:**

A real-time clinical decision support system (RTCDSS) with interactive diagrams enables clinicians to instantly and efficiently track patients' clinical records (PCRs) and improve their quality of clinical care. We propose a RTCDSS to process online clinical informatics from multiple databases for clinical decision making in the treatment of prostate cancer based on Web Model-View-Controller (MVC) architecture, by which the system can easily be adapted to different diseases and applications.

**Methods:**

We designed a framework upon the Web MVC-based architecture in which the reusable and extractable models can be conveniently adapted to other hospital information systems and which allows for efficient database integration. Then, we determined the clinical variables of the prostate cancer treatment based on participating clinicians' opinions and developed a computational model to determine the pretreatment parameters. Furthermore, the components of the RTCDSS integrated PCRs and decision factors for real-time analysis to provide evidence-based diagrams upon the clinician-oriented interface for visualization of treatment guidance and health risk assessment.

**Results:**

The resulting system can improve quality of clinical treatment by allowing clinicians to concurrently analyze and evaluate the clinical markers of prostate cancer patients with instantaneous clinical data and evidence-based diagrams which can automatically identify pretreatment parameters. Moreover, the proposed RTCDSS can aid interactions between patients and clinicians.

**Conclusions:**

Our proposed framework supports online clinical informatics, evaluates treatment risks, offers interactive guidance, and provides real-time reference for decision making in the treatment of prostate cancer. The developed clinician-oriented interface can assist clinicians in conveniently presenting evidence-based information to patients and can be readily adapted to an existing hospital information system and be easily applied in other chronic diseases.

## Background

In clinical practice, clinicians encounter a number of common problems when it comes to improving the quality of clinical treatments as follows: (1) clinicians may take several hours, or even a couple of days, to review patients' clinical records (PCRs) but only have a few minutes to explain their opinions to patients based on their records; (2) patients typically find it difficult to understand their condition since clinicians may only be able to explain the disease adequately using written descriptions; (3) although the traditional clinical decision support system (CDSS) is computerized, in many clinics it may not have online capability; (4) many commercial utilities provide computational tools but real-time analysis is not available unless the required modules are reusable or extractable. Therefore, many clinicians are in need of an expandable CDSS with an interactive diagrammed interface which can be used as an effective tool to efficiently evaluate instant PCRs and to make clinical decisions.

The research indicates that publicly released clinical evidence data seems to improve patient care quality at the hospital level [1]. Hence, a computerized clinical data analysis and information technology (IT)-based decision support system may be of value in decreasing workflow and data collection errors in order to improve communication with patients and enhance patient safety. Many studies have demonstrated that the consequences of errors in medical care were reduced by the use of computer-based CDSS in the provision of care in terms of clinician performance and patient outcome. Moreover, the results of several studies have shown improvements for drug dosing, preventive care, and other aspects of medical care with the use of computer-based CDSS, but their use in diagnosis has, to date, been less convincing [2-4]. Thus, a clinician-oriented interface with real-time analysis may be the key to improving accuracy and efficiency of the CDSS to meet hospitals' needs. Numerous CDSSs have been developed over the years for a variety of clinical approaches, such as the Web-based consultation library with evidence-based clinical literature, which can be searched and accessed remotely [5], the clinical decision model for integration with other clinical systems [6], and the initial framework of electronic patient self-assessment for healthcare awareness in cancer survivorship progress [7]. A platform with a flexible framework design which is capable of satisfying clinical requirements for diverse disease treatments is therefore needed.

Thus, we proposed a real-time clinical decision support system (RTCDSS) upon a reusable framework that was built with the extractable process models which support online clinical informatics. In addition, we designed a clinician-oriented interface to help the clinician process instantaneous clinical data for decision making. The system was initially applied for use in prostate cancer treatment as a pilot study. It has been well established in the literature that prostate-specific antigen (PSA) level, Gleason grade, and clinical TNM (tumor/nodes/metastases) stage are essential for developing a treatment strategy for prostrate cancer. Watchful waiting, radiation therapy, and surgery are generally offered to men whose cancer remains within the prostate; whereas hormonal therapy and chemotherapy are often reserved for those whose disease has spread beyond the prostate. To facilitate these treatments, many studies have suggested analytical tools to assist clinicians in estimating the relative pretreatment parameters and for tracking the proper diagnostic guidelines on visualized interfaces [8-14]. From clinical data tracking to real-time decision making tools, the flexible Web-based CDSS with online evidence-based medicine progress is a growing trend in advanced clinical care.

In this study, we developed a RTCDSS with novel Web technologies to integrate PCRs and patient-report outcomes (PROs) in order to generate visualized diagrams and interactive guidelines. The framework can be readily adapted for use by many hospital information systems (HISs). Herein, reusable computation models are introduced in subsequent sections. The major schema of the system is addressed in the methods section and the method of integrating the necessary components is described in the development section. In the results and discussions sections, the processes by which the system obtains the online pretreatment parameters and implements them for real-time decisions at clinic visits are described.

## Methods

The scope of the proposed system include: (a) to allow for flexible adaption of the functionality for heterogeneous HISs through the Internet, (b) to instantly present online PROs and PCRs for prostate cancer patients, and (c) to provide a user-friendly clinician-oriented interface for clinicians. We thus used the model-view-controller (MVC) design pattern with Web services to create a MVC-based architecture. The concept of this design pattern was first made available by Gamma *et al*. [15] who introduced 23 patterns associated with creational, structural and behavioral models of software design to process recurrent elements. The MVC essentially creates a hybrid of three of them: the strategy, observer, and composite patterns. It also divides system responsibilities into three parts: the model, which maintains program data and logic; the view, which provides a visual presentation of the model; and the controller, which processes user input and makes modifications to the model. With this architecture, the framework allows for reusable components to be applied in an expandable system and reduces the development complexity of Web-based applications. The framework of the proposed RTCDSS includes affiliated models, views and controllers for clinical informatics and implements Web services for online analytical process (OLAP).

### OLAP upon MVC-based Architecture

**Figure 1:**
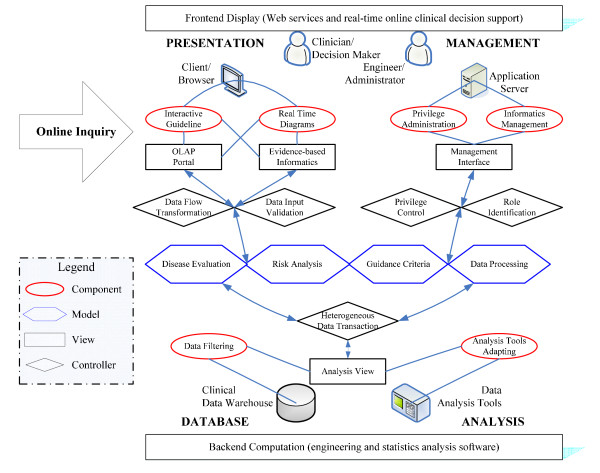
**Infrastructure of the RTCDSS upon MVC-based architecture**.

The built-in elements within the framework should be reusable and extractable to enable clinical analysis and decision support for clinical cares which includes: a) instantaneous disease evaluation, b) risk analysis, and c) treatment guidance. For these tasks, we designed the infrastructure of MVC-based architecture, shown in Figure 1, so that it involves presentation, management, analysis, and database tiers.

#### (i) Models

The models are categorized into four main groups: disease evaluation, risk analysis, treatment guidance, and data processing models. They are denoted by hexagonal blocks, in which the first three models are related to clinical data computation while the last one represents the other IT modules. The disease evaluation model primarily contains modules to retrieve clinical variables, calculate pretreatment parameters, and evaluate PROs and PCRs. The risk analysis model drives algorithms to analyze clinical variables and parameters, identify risk indicators and criteria, and so on. The guidance criteria model enables the generation of evidence-based diagrams, online guidance and decision support. The rest of the IT-related modules such as clinical data conversion, database connection, and graphical display, are included in the data processing model.

#### (ii) Views

The views denoted by the rectangles can implement the clinician-oriented interface directly with the OLAP portal and the evidence-based informatics for clinicians at the presentation tier. Similarly, the view of management interface support provides IT engineers with security administration at the management tier. Meanwhile, researchers can take care of all clinical data through the analysis view at the analysis and database tiers. Based on this design, these views are behind the major components of each tier denoted in the ellipse blocks such as real-time diagrams, interactive guidelines, privilege administration, informatics management, data filtering and data analysis tools.

#### (iii) Controllers

The controllers denoted by the rhombus support interactions among the models and views within the infrastructure. At the presentation tier, the controllers process data flow transformation and data input validation when the clinicians begin online inquiries. At the management tier, the privilege control and role identification are required when the engineers are conducting system maintenance. Meanwhile, the clinical data at the back tiers of analysis and database are coordinated by heterogeneous data transaction.

Based on this MVC-architecture, the online clinical informatics can be achieved by the OLAP mechanism. The OLAP is utilized as a decision support platform since it supports efficient online functionalities with computation algorithms upon the data warehouse [16]. For example, the clinician is a decision maker who represents the presentation tier which performs the components of the interactive guideline and real-time diagrams in the clinic. These components present online clinical informatics with the views of "OLAP portal" and "evidence-based informatics" by executing the computation models through the controllers of "data flow transformation" and "role identification". The rest of the object relationships may be deduced by comparing with the other tiers. Based upon this framework, all objects are independent but enable reciprocal supports through Web services. The proposed system was constructed by using Java™ technology to provide a clinician-oriented interface in a Web browser for real-time online decision support.

### Management of Distributed Database

The OLAP needs to process diverse clinical data within a variety of systems, thus two types of data flows should be considered: distributed database (DDB) management and extensible markup language (XML) schema. The DDB management coordinates the related PROs and PCRs of prostate cancer data that are stored in various systems. The architecture supports a virtual and centralized database to aggregate data from multiple databases. It provides transparent data transaction that allows users to alternate between sets of data as if there is an autonomous database operating independently of levels of distribution or heterogeneity. Meanwhile, the transportable XML documents contain a tree structure with hierarchical node elements that records data within the local server. Thus, we can parse subsequent nodes from XML files to retrieve the data. Some online data validations only request simple criteria instead of frequent database transaction. Hence, the XML schema is used for accessing distributed light-weight data through Web services.

To enhance performance of data transaction, the approach retains heavy-mass data necessary for routine query within the database server, (e.g., clinical variables, PROs and PCRs) and accesses lightweight data for online analysis in the Web server, (e.g., decision support criteria, treatment guidelines). This design takes load balance into consideration which is an important system performance factor when the data are spread across multiple Web servers. By using XML schema, the data for decision support are transformed into Web services documents in the Web server. Meanwhile, masses of clinical data are analyzed at the backend and provide expert opinions for feedback to the data warehouse. Therefore, complex data queries are not saved on the Web server site but instead are left on the database servers. The data transaction can then be done smoothly for efficient workflows on both the Web and database servers.

#### Development

While developing the proposed system, we first considered the requirements of online clinical informatics for prostate cancer treatments. We then discovered the framework of the RTCDSS to enable real-time decision making with heterogeneous data computation. Participating clinicians suggested using clinical variables below for long-term tracking and we applied statistical algorithms to create computation models for analyzing the pretreatment parameters and providing feedback of expert opinions.

### System Requirements for Decision Support

Prostate cancer is known to occur when genetic mutations of the prostate take place which then causes cells to begin multiplying out of control. Local invasion of tumors can lead to urethral obstruction and even renal failure while they spread to the bones and lymph nodes [17]. Among urologic malignancies, prostate cancer has greatly benefited from the discovery of a tumor marker and disease staging. The combination of treatments and serum PSA, particularly the initial PSA after a treatment, is the most useful clinical information for detecting, staging, and monitoring prostate cancer patients when assessing the risk of prostate cancer [18-21]; Disease stage, based on the TNM system, which includes the size of the tumor, the number of involved lymph nodes, and the presence of any other metastases, indicates how far the cancer has spread for defining prognosis and selecting therapies. Herein, the clinical variables were retrieved from heterogeneous databases of a HIS through different networks via secure interfaces adapted to the proposed system.

#### (i.) PSA Level

The presence of prostate diseases is the most important factor affecting serum levels of PSA [22,23]. Many studies have made efforts to evaluate other thresholds to maximize the positive biopsy rate of PSA-based screening [24-27]. The PSA-related parameters including PSA density (PSAD), PSA velocity (PSAV) and PSA doubling time (PSADT) are considered to improve diagnostic accuracy of PSA.

A direct relationship between PSAD and the likelihood of cancer has been documented [28], and higher PSA densities may be found among groups of men with positive biopsies compared with men with negative biopsies [29]. PCRs can be filtered to rank high risk patients who have relatively smaller prostate volumes when a constant number of biopsies are obtained.

PSAV is the rate of change in serum PSA. A rate in excess of 0.75 ng/mL per year is a significant indicator of the presence of prostate cancer and some studies have suggested increasing the cut point to more than 2 ng/mL of PSA per year for prediction of prostate cancer [30,31]. We can estimate PSAV by applying linear regression to PSA data. A linear equation for arbitrary PSA (P_i_) with respect to time (T_i_) can then be formulated. The estimator P_i _at time T_i _can be denoted by the equation, P_i _= initial PSA + PSAV * T_i_. In practice, T_i _can be counted by days, months or years.

PSADT is denoted as the duration when the logarithm of PSA doubles and has been evaluated in patients with a rising PSA after local treatment with radiation therapy [32]. In order to obtain the PSADT value, we can substitute the regression equation of PSAV into the half-logarithmic coordinate of ln(P_i_) versus T_i_, and a straight line is obtained to calculate doubling PSA at doubling time T_D_. Therefore, if two arbitrary PSAs (P_1 _and P_2_) are measured at time T_1 _and T_2_, respectively, T_D _can be estimated as ln(2*P_1_) is interpolated. Similarly, T_i _can be counted by days, months or years.

#### (ii.) TNM stage

The well-known TNM classification system generally evaluates the size of the tumor (T) by four stages, the extent of involved lymph nodes (N) by two stages, and any metastasis (M) by two stages. In this study, version 6 of the TNM system published by the American Joint Committee for Cancer (AJCC) and the International Union against Cancer (UICC) in 2002 was used.

### Evaluation Criteria of Expert Opinions

The significant pretreatment parameters described below can be used to track prostate cancer patients periodically and can be applied in the development of computational models.

#### (i) Gleason grade and Partin table

The Gleason grading system is the most common scheme for classifying the histological grading of prostate cancer [33]. The predominant pattern that occupies the largest area of the specimen is given a grade between 1 and 5. This number is then added to the grade assigned to the second most dominant pattern; thus, a Gleason sum may be between 2 and 10. Partin tables include primary tumor stage, serum PSA level, and Gleason grade to determine the probability of having a final pathologic stage based on logistic regression analyses for all 3 variables combined [34,35]. In this study, the system applied the Partin table used by the National Comprehensive Cancer Network (NCCN).

#### (ii) Risk evaluation criteria

Risk evaluation criteria of prostate cancer is constructed on the basis of large numbers of patients who have undergone radical prostatectomy to aid in the precise prediction of pathologic stage by using multiple clinical parameters as accurate predictors of both cancer extent and long-term outcomes after treatment of the primary tumor [36]. We adopted the criteria suggested by D'Amico *et al*. [37] to stratify patients into low-risk, intermediate-risk, and high-risk disease, and to summarize the failure status, as shown in Table 1. The criteria define the risk factors for three levels of risk for three conditions, i.e., (a) prostate-therapy PSA failure at 5 years, (b) PSA failure-free survival at 5 and (c) 10 years. In the table, for example, condition (a) presents three levels of risk less than 25%, between 25% and 50%, and greater than 50% as the risk factors match the criteria.

**Table 1:** Risk Evaluations for Prostate Cancer

Risk group	Risk factors	Risk (a, b, c)%
Low	T1c or T2a and PSA <= 10 ng/ml and Gleason score <= 6	(< 25, 85, 83)

Intermediate	T2b or Gleason score = 7 or PSA > 10 and <= 20 ng/ml	(25-50, 60, 46)

High	T2c or PSA > 20 ng/ml or Gleason score >= 8	(> 50, 30, 29)

a. Post-therapy PSA failure at 5 yrs; b. PSA failure-free survival at 5 yrs;

c. PSA failure-free survival at 10 yrs

Based on the correlations of these pretreatment parameters with the true extent of disease, the RTCDSS can integrate the clinical data and expert opinions available for clinicians to determine the likelihood of disease progression and predict the pathologic stage.

### Framework Integration with Clinical Data

In order to develop the RTCDSS efficiently, we considered an open source framework to integrate heterogeneous clinical data with required components above.

#### (i) Open Source Framework

**Figure 2:**
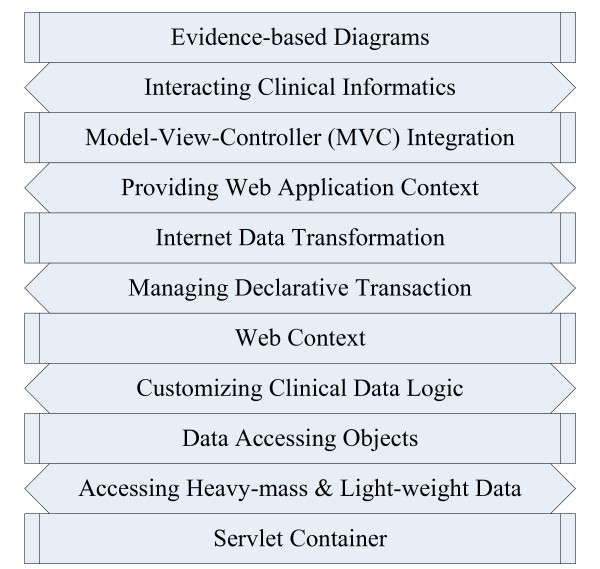
**Clinical data flow throughout the integration framework**.

The Spring™framework, which is well-known as a business to business (B2B) open source framework in the IT industry, was used to construct the platform for online computation and distributed data management in a variety of HIS. The data flow follows throughout the stack chart shown in Figure 2 to control the model objects with the necessary procedure. Based on the chart, when PCR data arrive at the object of the servlet container, the framework begins the processes of assessing heavy-mass and lightweight data and customizing the clinical data logic until the object of Web context is fulfilled with declared data. The Web services are then able to manage declarative transaction and complete the object of Internet data transformation for provision of the Web application context. Then, on the next stack, one object may drive the disease evaluation model with the MVC pattern, and another object may take the risk analysis model for inheriting a part of the clinical data. Thus, they require the object of MVC integration to yield the clinical informatics. Finally, the clinicians can present evidence-based diagrams by interacting with the clinical informatics. This dataflow allows these objects to control heavy and light clinical data access for balancing loads.

#### (ii) Heterogeneous Data Integration

**Figure 3:**
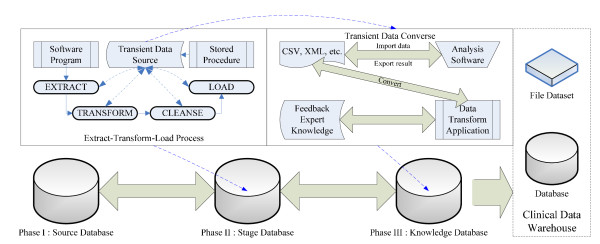
**The three-stage integration of clinical data in the RTCDSS**.

To incorporate pretreatment parameters with PCRs and PROs for online analysis, we generated a clinical data warehouse to support expert opinion feedback for decision making. Figure 3 presents a three-stage data progress flow from various source data to the data warehouse. The source database for clinics would be unified by the extract-transform-load (ETL) process of software program to extract, transform, cleanse and load the transient data source with the stored procedure into stage database. The pretreatment parameters were further manipulated with transient data by analysis applications to yield expert opinions and feedback to the knowledge database behind the clinical data warehouse. We used three primary controller modules, which are dynamic views, stored procedures, and triggers, in the database software to automatically perform data transactions while integrating diverse data. The ETL process conducts the data filtering function, as shown in Figure 1, while the data transformation application can be adapted to employ analysis tools, such as the hazard model, the survival model, or other statistical algorithms by SAS™or Matlab™for feedback of expert opinions.

Using the developed components above, the proposed framework can provide clinicians with the ability to immediately assess pretreatment parameters in addition to collating PCRs and PROs via online informatics, which better enables them to inform and educate patients during clinic visits.

## Results

The proposed MCV-based RTCDSS contains 4 tiers, while the groups of three models and two views within the presentation tier support clinicians' decision making. The three primary models are disease evaluation, guidance criteria, and risk analysis, which are created by the feedback of clinicians' expert opinions. The diseases evaluation model includes PCRs, such as PSA level, Gleason grade, TNM stage, as well as PROs, such as real-time evaluation for quality of life. We employed pretreatment parameters, such as PSAV, PSAD, Partin tables, as the guidance criteria for online clinical informatics of prostate cancer. The risk analysis model was then used to compute the informatics of disease evaluation and guidance criteria. The results contain two views, which are the "OLAP portal" and the "evidence-based informatics," to provide clinician-oriented interface with graphical diagrams that may aid the interaction between clinicians and patients during discussion of treatment options. The proposed framework was practiced in the urological cancer department of China Medical University Hospital (CMUH) in Taichung, Taiwan, by incorporating campus and hospital networks using heterogeneous database management. The necessary data resources were extracted and filtered from the prostate cancer database of CMUH. Patients who received treatments for prostate cancer were enrolled in the pilot study which was approved by the institutional review board (IRB). The design of the clinician-oriented interface and its developed functions was evaluated with regard to how well it helped clinicians interact with patients and provide efficient clinical care.

**Figure 4:**
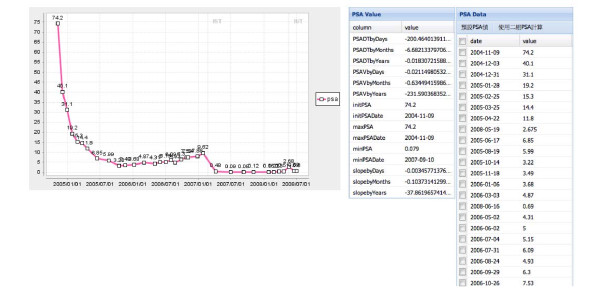
**A screen shot of interactive guidance that the clinicians can use to select the historical PSA data online to aid decision making**.

A. Disease evaluation of the PSA level - Figure 4 shows the PSAV and PSADT values with PSA baseline while the clinician enters the patient's ID and selects an arbitrary time interval. The real-time diagram shows the disease information of the patient's PSA level throughout different treatments. It can be seen that the system retrieved the patient's data from PCRs and listed related pretreatment parameters for an overview of the patient's disease history. The baseline of PSA was completely plotted during different treatment cycles with significant points (such as the initial PSA) of note. The clinicians could evaluate both the PSAV and PSADT as two algorithms were used by either using the average value of listed PSAs or choosing two specific PSA markers.

**Figure 5:**
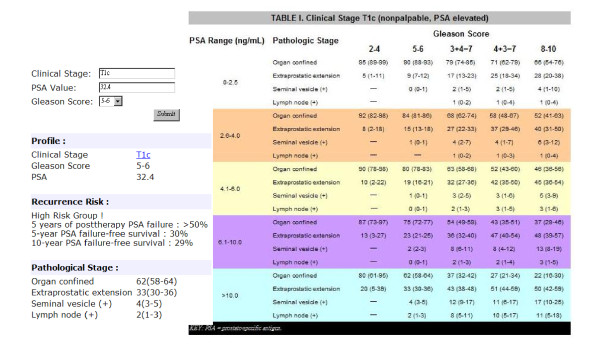
**A screen shot of real-time decision support that the clinician can use to evaluate the recurrence risk and the Partin table online by flexibly adjusting clinical data**.

B. Risk analysis with Partin tables - Using the Partin tables module in the system, the clinician can easily find and input pretreatment parameters such as PSA, Gleason score, and a clinical stage to determine the risk percentage shown in Figure 5. In the example, the clinician entered the risk factors of the selected patient which were T1c, 32.4, and 5-6 for TNM stage, PSA value and Gleason score, respectively. The system then estimated the recurrent risk percentage and classified patient into the high risk group immediately while the pathological stage was displayed with the Gleason score on a new web page of the NCCN Partin table for reference. The risk evaluation in Table 1 also represents the feedback of expert opinions that may be adjusted by advance statistics.

**Figure 6:**
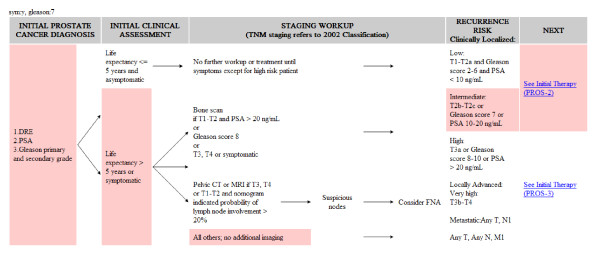
**A screen shot of interactive guideline that can highlight a suitable prostate cancer treatment flow based on a patient's clinical data**.

C. Interactive treatment guidance - Figure 6 illustrates the guideline flowchart by query pretreatment parameters from PCRs and proposes the treatment phase based on the criteria for decision making. According to the presented example, PSA was 82.35 ng/ml, the clinical stage was T2cN0M0, Gleason Score was 4+3, life expectancy was 15 years, and lymph node involvement was 38%, with asymptomatic therapy. The pink region instantaneously highlighted the therapeutic steps for reference while the pathological information of the selected patient was entered. By providing a comparison with the non-highlighted steps on the overview of guideline flow, the flowchart allows the clinicians to identify the current stage and see what the next step is.

## Discussions

Due to restrictions related to hospital management and security policy, the system could not be used by all clinical care staffs or be used beyond the hospital network at all times. On this phase, we therefore focused on the core system development and initial clinical applications. Prior to this study, the participating clinicians would typically study patients' data for several hours before explaining the disease conditions to their patients. During the study period, the clinicians were able to use real-time online diagrams to help them make clinical decisions and evaluate treatment effectiveness. The biggest benefits of the system would likely be enhanced clinical care for patients, better identification of optimal treatment options, and increased efficiency in clinics. The main advantages of the developed system are described in more detail below.

### A. Online informatics for clinicians

The greatest advantage conferred by this system is its ability to assist in the treatment of chronic diseases that can be periodically tracked using the specific clinical variables. The interface of online informatics for prostate cancer patients displays their PSA-related data associated with statistical modules to provide categories of diagnostic information. Clinicians are able to identify patients' health conditions directly with respect to treatments through the instant diagrams. The clinicians in the study reported that the RTCDSS saved them hours, even several days, of analysis by providing instant computation of the relevant parameters. This study confirms that the system can help clinicians quickly and accurately identify treatment options, make the correct decision, and save time that was previously wasted.

### B. Quality of treatment by system execution

The online guideline suggests the surgery treatment for early-stage and younger patients as opposed to radiotherapy for severe-stage or elder patients. If a patient's PSA is less than either 0.2 ng/ml or 2 ng/ml after the surgery or radiotherapy, respectively, then the treatment is counted as successful. In this study, 95 of prostate cancer patients were selected for the pilot study because their initial PSA data were recorded before the system was installed. They were more easily convinced by evidence-based diagrams with risk evaluation before accepting the treatments. Correspondingly, 61 were suggested for surgery and 34 for radiotherapy. They were also tracked after treatments for various periods, 12 months at most. Table 2 shows the information of treatments resulted in better control for patients who were cured by either surgery or radiotherapy. In general, the successful rates for both treatments reached more than 80%. Most of the patients' PSA values improved after the treatments, which confirms the treatment quality.

**Table 2:** Percentage improvements in patients' PSA after surgery and radiotherapy treatments

	months after treatment	1	3	6	9	12
Treatment		(no. of successful treatments/patient number)				
Surgery	PSA < 0.2 ng/ml	29/47 (62%)	46/53 (87%)	34/44 (77%)	34/43 (79%)	28/33 (85%)
	lower than pre-treatment	46/47 (98%)	53/53 (100%)	44/44 (100%)	42/43 (98%)	33/33 (100%)

Radiotherapy	PSA < 2 ng/ml	10/27 (37%)	15/30 (50%)	17/28 (61%)	19/24 (79%)	18/21 (86%)
	lower than pre-treatment	25/27 (93%)	26/30 (87%)	24/28 (86%)	20/24 (83%)	18/21 (86%)

### C. Improvement in the clinician-patients relationships

Several studies in chronic diseases suggest that feedback of health status data may facilitate communication between patients and clinicians and enhance patients' care [38]. In this study, clinicians showed patients their PSA-related trends via the charts, as shown in Figure 4 and explained to them the predicted potential disease risk which was presented to them in a table, as shown in Figure 5. Thus, the clinician could refer to the interactive guideline in Figure 6 while offering the suggestions, ordering the proper treatment, and tracking the follow-up conditions. *The developed system enhances clinicians' awareness of their patients' data with reliable and predictive information related to prostate cancer treatments through the real-time computation*. Data quality is hence ensured by the automatic transportation procedure inherent in the system which minimizes manual mistakes. It confirms the developed RTCDSS has the capacity to improve clinician-patient relationships.

In the future, due to the flexibility and expandability of the system, real-time data can be updated to incorporate developed decision support functions with prediction models such as well-known nomograms, which may help patients and their treating physicians make informed decisions based on the probability of a pathologic stage, the individual patient's risk tolerance, and the values they place on the various potential outcomes [39]. The system will aid the rational selection of patients to undergo definitive therapy.

## Conclusions

In this study we developed a novel real-time clinical decision support system (RTCDSS) which was capable of integrating data from numerous databases for practical use by clinicians to provide immediate visual feedback, facilitate decision-making, and to improve quality of care. Such databases may include patients' clinical records, patient-reported outcomes, clinical variables, and physicians' practice guidelines. The proposed system was applied for online clinical informatics of prostate cancer as a pilot study. The system conferred the following advantages: (1) clinicians could clearly explain health conditions to patients by visualized clinical variables and pretreatment parameters; (2) patients were more easily convinced by evidence-based diagrams before accepting the risk evaluation of treatments and the treatment quality was confirmed; (3) the design presents a clinician-oriented interface for real-time disease and risk evaluation while the interactive guidelines with treatment suggestions offer the clinician efficient online tools for instant decision making; (4) the proposed framework for prostate cancer treatment was constructed upon the MVC-based architecture that consists of reusable models, making it flexible and adaptable for use in many hospital information systems (HISs).

The results of this pilot study are related to prostate cancer. However, the Web Model-View-Controller (MVC) architecture can be readily applied in any traceable chronic disease, such as chronic pulmonary obstructive disease and asthma, as it allows for integration of a wide range of relevant data in real-time to facilitate decision-making and improve quality of care.

## List of Abbreviations

CDSS: Clinical Decision Support System; RTCDSS: Real-Time CDSS; MVC: Model-View-Controller; OLAP: OnLine Analytical Process; DDB: Distributed Database; XML: eXtensible Markup Language; HIS: Hospital Information System; B2B: Business to Business; PCR: Patients' Clinical Record; PRO: Patient-Reported Outcome; PSA: Prostate-Specific Antigen; PSAD: PSA density; PSAV: PSA velocity; PSADT: PSA doubling time; TNM: primary Tumor, lymph Node, distance Metastasis.

## Competing interests

The authors declare that they have no competing interests.

## Authors' contributions

HCL is the corresponding author who conceived of the study, and contributed to its design, development and coordination and drafted the manuscript. HCWparticipated in the clinical practice integration and was the consultant for the knowledge of prostate cancer. CHC interpreted the concept of the system prototype and helped revise the manuscript. All authors read and approved the final manuscript. TCL, WML and CYW collaborated to collect the clinical data and perform the statistical analysis.

## Authors' information

*Hsueh-Chun Lin*, PhD, (snowlin@mail.cmu.edu.tw, 886-4-22053366 ext. 6506, Fax: 886-4-22070429), Assistant Professor, Department of Health Risk Management, China Medical University.

*Hsi-Chin Wu*, M.D, wuhc@mail.cmu.edu.tw, Associate Professor, Department of Medicine, China Medical University.

*Chih-Hung Chang*, Ph.D., chchang@northwestern.edu, Associate Professor, Buehler Center on Aging, Health & Society and Department of Medicine, Feinberg School of Medicine, Northwestern University, and Adjunct Professor, Graduate Institute of Biostatistics, China Medical University.

*Tsai-Chung Li*, Ph.D., tcli@mail.cmu.edu.tw, Professor, Graduate Institute of Biostatistics & Biostatistics Center, China Medical University.

*Wen-Miin Liang*, Ph.D., wmliang@mail.cmu.edu.tw, Professor, Graduate Institute of Biostatistics & Biostatistics Center, China Medical University.

*Jong-Yi Wang Wang*, Ph.D., ericwang@mail.cmu.edu.tw, Assistant Professor, Department of Health Services Administration, China Medical University.

## Pre-publication history

The pre-publication history for this paper can be accessed here:

http://www.biomedcentral.com/1472-6947/11/16/prepub
